# Supplementary material for: New Insights into Arrestin Recruitment to GPCRs
Source: Int J Mol Sci. 2020 Jul 13;21(14):4949. doi: 10.3390/ijms21144949 (PMC7404097; doi:10.3390/ijms21144949)
Supplement: Supplementary file 1 [file ijms-21-04949-s001.pdf]

Supplementary Figure 1: List of plasmids

See Kriz et al [154] for a description of plasmid backbones.

Fig. 1: Direct Assay

AG10-B2AR-11S (#685)

AG10-GRPR-11S (#728)

AK1-SSTR2-11S (#762)

AA6-114-S-Arrestin (#940)

AA6-114-Arrestin-C (#943)

AG10-bArrestin1 (#691)

AG10-bArrestin2 (#692)

Figure 2: Indirect Assay

AG10-11S-CAAX (#680)

AK-SSTR2 (#515)

AK-GRPR (#517)

AG10-B2AR wt (#503)

AA6-114-S-Arrestin (#940)

AG10-114-bArrestin1 (#691)

AG10-114-bArrestin2 (#692)

Figure 2e: Stable cell lines

AA6-114-S-Arrestin (#940)

AK1-114-bArrestin1 (#683)

AK1-114-bArrestin2 (#684)

Figure 3: Comparison of different assay types

AG10-B2AR-11S (#685)

AG10-GRPR-11S (#728)

AK1-SSTR2-11S (#762)

AK1-114-bArrestin2 (#684)

Figure 4: C-tail truncations

AG10-GRPR-S346cut-11S (#962)

AG10-B2AR-11S S345X (#748)
AG10-114-bArrestin2 (#692)
AG10-114-bArrestin1 (#691)

Figure 5: Influence of expression levels
AK-SSTR2-11S (#762)
AK-B2AR-11S (#685)
AK-GRPR-11S (#728)
AK1-114-bArrestin2 (#684)
AG-CMV-GRPR-11S x DS-CMV-114-bArrestin1-mCh
AG-CMV-GRPR-11S x DS-PGK-114-bArrestin1-mCh
AG-CMV-GRPR-11S x DS-CMV-114-bArrestin2-mCh
AG-CMV-GRPR-11S x DS-PGK-114-bArrestin2-mCh
AG-PGK-GRPR-11S x DS-CMV-114-bArrestin1-mCh
AG-PGK-GRPR-11S x DS-PGK-114-bArrestin1-mCh
AG-PGK-GRPR-11S x DS-CMV-114-bArrestin2-mCh
AG-PGK-GRPR-11S x DS-PGK-114-bArrestin2-mCh

Figure 6: Baculovirus generation
AG10-114-bArrestin1 x DS2cx-11S-CAAX (#693)
AG10-114-bArrestin2 x DS2cx-11S-CAAX (#694)
AG10-b2AR-11S x DScx-114-bArrestin-1 (#781)
AG10-b2AR-11S x DScx-114-bArrestin-2 (#785)
AG10_114-bArrestin1 x DSZ2_11S-CAAX x DA2-SSTR2 (#905)
AG10_114-bArrestin1 x DSZ2_11S-CAAX x DA2-GRPR (#906)
AG10_114-bArrestin2 x DSZ2_11S-CAAX x DA2-B2AR (#907)
AG10_114-bArrestin2 x DSZ2_11S-CAAX x DA2-SSTR2 (#908)
AG10_114-bArrestin2 x DSZ2_11S-CAAX x DA2-GRPR (#909)

Supplementary Figure 2: Primers and gene blocks

Name	Sequence	Function
SapI-Barr1-back	GAT CGC TCT TCT TCA TCT GTT GTT GAG CTG TGG	Primer for amplification of β-Arrestin 1
BamHI-BArr2-for	GAT CGG ATT CAT GGG GGA GAA ACC CGG G	Primers for amplification of β-Arrestin 2
SapI-BArr2-for	GAT CGC TCT TCC TCA GCA GAG TTC ATC ATC ATA	
114-Arr2-for	GCTA GCTCTTC A ATG GTC ACG GGC TAC CGC CTG TTC GAG GAA ATC CTC GGG GGG TCT GGT GGC GGT GGG AGT GGG GGG TCT AGC AGT GGT GGA TCC ATG GGC GAC AAA GGG ACG CG	Split NanoLuc with linker and β-arrestin-1
11S-CAAX for	GTAC GCTCTTC G ATG GTG TT	Amplification of the 11S-CAAX gene block
11S-CAAX rev	CTAG GCTCTTC G TCA CAT GA	
Linker-11S for	GTAC GGA TCC GGG TCT GGT G	Amplification of the Linker-11S gene block
Linker-11S rev	CTAG GCTCTTC G TCA GGGCC	
huRhodopsin for	AGC TTG GTA CCG AGA TAT ACC ATG AAT GGC ACA GAA GGC	Amplification of human rhodopsin
huRhodopsin back2	GAT CGA TCG GAT CCG GCC GGG GCC ACC TGG CTC	
11S-CAAX	GTAC GCTCTTC G ATG GTG TTC ACT CTC GAA GAC TTT GTC GGG GAT TGG GAA CAA ACG GCT GCG TAT AAT CTG GAT CAA GTC CTG GAG CAA GGC GGG GTG TCA TCT CTG TTG CAG AAT CTC GCT GTC TCC GTA ACA CCC ATT CAG AGG ATT GTT CGG TCA GGC GAG AAT GCGCTG AAG ATT GAT ATT CAC GTA ATA ATA CCA TAT GAG GGG CTC TCT GCG GAT CAG ATG GCT CAA ATA GAA GAG GTG TTT AAA GTA GTT TAC CCG GTT GAT GAC CATCAT TTC AAG GTA ATC CTC CCC TAT GGA ACG CTG GTT ATC GAC GGG GTA ACC CCA AAC ATG CTC AAT TAC TTT GGG CGG CCC TAT GAA GGT ATA GCT GTC TTT GAC GGT AAG AAG ATC ACC GTA ACC GGC ACA CTT TGG AAT GGG AAC AAG ATT ATC GAC GAG AGA CTC ATC ACA CCT GAT GGA AGC ATG TTG TTT CGA GTG ACA ATA AAT AGT GGC AGC GCC GGC ACC ATG GCC AGC AAC AAC ACC GCC AGC GGC GGC AAG AAA AAG AAA AAG AAA AGC AAA ACC AAG TGC GTG ATC ATG TGA C GAAGAGC CTAG	
Linker-11S	GTAC GGA TCC GGG TCT GGT GGC GGT GGG AGT GGG GGG TCT AGC AGT GGT GGT ATG GTG TTC ACT CTC GAA GAC TTT GTC GGG GAT TGG GAA CAA ACG GCT GCG TAT AAT CTG GAT CAA GTC CTG GAG CAA GGC GGG GTG TCA TCT CTG TTG CAG AAT CTC GCT GTC TCC GTA ACA CCC ATT CAG AGG ATT GTT CGG TCA GGC GAG AAT GCG CTG AAG ATT GAT ATT CAC GTA ATA ATA CCA TAT GAG GGG CTC TCT GCG GAT CAG ATG GCT CAA ATA GAA GAG GTG TTT AAA GTA GTT TAC CCG GTT GAT GAC CAT CAT TTC AAG GTA ATC CTC CCC TAT GGA ACG CTG GTT ATC GAC GGG GTA ACC CCA AAC ATG CTC AAT TAC TTT GGG CGG CCC TAT GAA GGT ATA GCT GTC TTT GACGGT AAG AAG ATC ACC GTA ACC GGC ACA CTT TGG AAT GGG AAC AAG ATT ATC GAC GAG AGA CTC ATC ACA CCT GAT GGA AGC ATG TTG TTT CGA GTG ACA ATA AAT AGT TGA GGGCCC TGA C GAAGAGC CTAG	
